# The role of genetic polymorphisms in the sulfation of pregnenolone by human cytosolic sulfotransferase SULT2B1a

**DOI:** 10.1038/s41598-024-56303-y

**Published:** 2024-04-05

**Authors:** Eid Alatwi, Ahsan F. Bairam

**Affiliations:** 1 Department of Pharmacology, College of Pharmacy, Jouf University, Sakaka, Al-Jouf Region Saudi Arabia; 2 Department of Pharmacology and Experimental Therapeutics, College of Pharmacy and Pharmaceutical Sciences, University of Toledo Health Science Campus, Toledo, OH 43614 USA; 3 Department of Clinical Pharmacy, College of Pharmacy, University of Kufa, Kufa Street, Najaf, 540011 Iraq

**Keywords:** Pregnenolone, Sulfation, SULT2B1a, SNPs, Biochemistry, Genetics, Physiology, Diseases, Endocrinology, Gastroenterology, Medical research

## Abstract

Pregnenolone is a key intermediate in the biosynthesis of many steroid hormones and neuroprotective steroids. Sulfotransferase family cytosolic 2B member 1 (SULT2B1a) has been reported to be highly selective to sulfate pregnenolone. This study aimed to clarify the effect of missense single nucleotide polymorphisms (SNPs) of the human *SULT2B1* gene on the sulfating activity of coded SULT2B1a allozymes toward Pregnenolone. To investigate the effects of single nucleotide polymorphisms of the *SULT2B1* gene on the sulfation of pregnenolone by SULT2B1a allozymes, 13 recombinant SULT2B1a allozymes were generated, expressed, and purified using established procedures. Human SULT2B1a SNPs were identified by a comprehensive database search. 13 SULT2B1a nonsynonymous missense coding SNPs (cSNPs) were selected, and site-directed mutagenesis was used to generate the corresponding cDNAs, packaged in pGEX-2TK expression vector, encoding these 13 SULT2B1a allozymes, which were bacterially expressed in BL21 E. coli cells and purified by glutathione-Sepharose affinity chromatography. Purified SULT2B1a allozymes were analyzed for sulfating activities towards pregnenolone. In comparison with the wild-type SULT2B1a, of the 13 allozymes, 11 showed reduced activity toward pregnenolone at 0.1 µM. Specifically, P134L and R259Q allozymes, reported to be involved in autosomal-recessive congenital ichthyosis, displayed low activity (1–10%) toward pregnenolone. The findings of this study may demonstrate the impact of genetic polymorphism on the sulfation of pregnenolone in individuals with different *SULT2B1* genotypes.

## Introduction

Pregnenolone, the precursor of steroid hormones, can be synthesized from cholesterol in various organs such as the adrenal cortex, other peripheral steroidogenic tissues, and the brain under the action of cytochrome P450, CYP11A1^1^. Pregnenolone serves as an intermediate in the biosynthesis of many steroid hormones, including progestogens, androgens, estrogens, glucocorticoids, mineralocorticoids, and dehydroepiandrosterone (DHEA)^2^. Pregnenolone and DHEA function as sex hormone precursors and neuroprotective steroids^3^, and their sulfate derivatives display excitatory cellular actions that are important in learning and memory during cognitive aging^4^. In particular, the sulfoconjugated form of pregnenolone, pregnenolone sulfate, is involved in enhancing cognitive abilities and memory through gamma-aminobutyric acid A receptor and N-methyl-D-aspartate receptor^5^. This neuroactive metabolite has been reported to be highly detected in the human brain^6^. Besides, steroid hormone sulfates can also be deconjugated and exert their steroid function in target tissues such as the breast, prostate, and testis^7^.

Steroid hormone sulfates are synthesized by the hydroxysteroid sulfotransferases (SULT2A1 and SULT2B1), which catalyze the transfer of a sulfonate group from 3’-phosphoadenosine-5’-phosphosulfate (PAPS) to the 3-hydroxy group of steroids^8^. Sulfation reactions play essential roles in humans and other mammals, and constitute an important conjugation pathway for metabolizing xenobiotic compounds^9^. In cytosol, inorganic sulfate is conjugated with adenosine monophosphate by adenosine triphosphate (ATP) sulfurylase to form adenosine 5’-phosphosulfate (APS), which is subsequently phosphorylated under the action of APS kinase to form PAPS^10^. There are 13 known cytosolic sulfotransferase genes (*SULTs*) in humans, of which *SULT2* family members (SULT2A1 and SULT2B1) have been shown to be responsible for the sulfation of steroids^11^. The SULT2B1 subfamily consists of two members, SULT2B1a and SULT2B1b, both of which are encoded by the same gene^12^. SULT2B1a and SULT2B1b differ only in their amino terminus. Both SULT2B1a and SULT2B1b are capable of sulfating pregnenolone; SULT2B1a favors more strongly pregnenolone as a substrate^13^. In addition, SULT2B1a has been reported to be able to sulfate DHEA^14^ and the 3α- and 3β-hydroxyl groups present in sterols and bile acids^15^.

Genetic polymorphisms of the *SULT2B1* gene have been reported to be associated with several diseases, and it has recently been reported that SULT2B1 gene polymorphisms affect the sulfation of pregnenolone by the SULT2B1b variant^16^. A total of 10 SULT2B1b allozymes showed decreased sulfating activity toward pregnenolone, implicating that inter-individual variations in the specific activity of SULT2B1b may result in different steroid hormone homeostasis. A noteworthy question, however, is whether the sulfating activity of another SULT2B1 variant, SULT2B1a, can be influenced by nonsynonymous coding SNPs (cSNPs) in *SULT2B1*, thus leading to distinct physiological and pathological effects related to steroid metabolism in different individuals.

Since SULT2B1a plays a significant role in the homeostasis and metabolism of endogenous compounds, this study hypothesized that *SULT2B1* coding SNPs may lead to SULT2B1a allozymes having different sulfating activities toward pregnenolone. In order to verify the hypothesis, a systematic search was performed on *SULT2B1* SNPs deposited in several SNP databases. In total, 13 nonsynonymous cSNPs in *SULT2B1a* were selected and investigated based on the potential importance of their amino acid substitutions. The corresponding SULT2B1a allozymes were generated, expressed, and purified, and their sulfating activities toward pregnenolone were examined with three different substrate concentrations.

## Materials and methods

### Materials

Pregnenolone, adenosine 5′-triphosphate (ATP), 4-(2-hydroxyethyl)-1-piperazineethanesulfonic acid (HEPES), Trizma base, and dithiothreitol (DTT) were from Sigma-Aldrich (St. Louis, MO, USA). PrimeSTAR^®^ Max DNA polymerase was a product of Takara Bio (Mountain View, CA, USA). Oligonucleotide primers were synthesized by Eurofins Genomics (Louisville, KY, USA). *Dpn I* and the DNA ladder were products of New England Biolabs (Ipswich, MA, USA). QIAprep^®^ Spin Miniprep Kit was from QIAGEN (Germantown, MD, USA). Glutathione Sepharose was a product of GE Healthcare Bio-Sciences (Pittsburgh, PA, USA). Cellulose TLC plates and Ultrafree-MC 5000 NMWL filter units were from EMD Millipore (Billerica, MA, USA). X-ray films were sourced from Products International Corporation (Mt Prospect, IL, USA). Ecolume scintillation cocktail was purchased from MP Biomedicals, LLC. (Solon, OH, USA). Carrier-free sodium [^35^S] sulfate was from American Radiolabeled Chemicals (St. Louis, MO, USA). [^35^S]PAPS was synthesized from adenosine triphosphate (ATP) and carrier-free [^35^S]sulfate using recombinant human bifunctional ATP sulfurylase/ adenosine 5’-phosphosulfate (APS) kinase. The [^35^S]PAPS synthesized was adjusted to the required concentration and specific activity of 15 Ci/mmol at 1.4 mM by the addition of unlabeled PAPS^17^. All other chemicals were of the highest grade commercially available.

## Methods

### Identification and analysis of human SULT2B1 SNPs

Five online databases, U.S. National Center for Biotechnology Information (NCBI) (RRID: SCR_006472), Ensembl Variation (RRID: SCR_001630) database, the Research Collaboratory for Structural Bioinformatics Protein Data Bank (RCSB PDB) (RRID: SCR_012820), Online Mendelian Inheritance in Man (OMIM) (RRID: SCR_006437), and The Universal Protein Resource (UniProt) (RRID: SCR_002380), were systematically searched to identify the sequence variations of the human *SULT2B1* gene. Human *SULT2B1* SNPs were identified and categorized based on their location in the gene into coding SNPs (synonymous, nonsense, and non-synonymous (missense)), non-coding SNPs (introns, 5’-untranslated region (5’UTR), and 3’-untranslated region (3’UTR)).

### Preparation of cDNAs encoding different human SULT2B1a allozymes, expression, and purification of recombinant SULT2B1a allozymes

Polymerase chain reaction (PCR) was performed to generate cDNAs encoding SULT2B1a allozymes using specific sense and antisense mutagenic primers and PrimeSTAR^®^ Max DNA polymerase. The PCR-amplified wt-SULT2B1a cDNA packaged in pGEX-4 T-2 prokaryotic expression vector was used as a template. PCR amplification conditions consisted of 12 cycles of 30 s at 94 °C, 1 min at 55 °C, and 7 min at 72 °C. The final PCR reaction mixtures were incubated for one h at 37 °C with *Dpn* I to digest the wild-type template. Upon digestion of the wild-type template, pGEX-4 T-2 plasmids containing mutated SULT2B1a cDNAs were transformed into NEB 5-alpha *E. coli* competent cells and then extracted from the cells. The individual mutated SULT2B1a cDNA, verified the nucleotide sequences^18^, was then transformed into competent BL21 *E. coli* cells. The transformed cells were grown in LB medium containing 100 μg/mL ampicillin, and 0.1 mM Isopropyl-β-D-thiogalactopyranoside (IPTG) was supplied at OD600 nm ∼0.5. After overnight incubation at 25 °C, cells were collected and resuspended in 20 mL ice-cold lysis buffer (10 mM Tris–HCl, pH 8.0, 150 mm NaCl, and 1 mM EDTA) and homogenized using an Aminco French press. The crude homogenates were centrifuged at 10,000 × *g* for 20 min at 4 °C, and the supernatants were individually fractionated with glutathione sepharose resin. The resin, washed with lysis buffer, was treated with thrombin digestion buffer (50 mM Tris–HCl, pH 8.0, 150 mM NaCl, and 2.5 mM CaCl_2_) containing 3.5 unit/ml bovine thrombin for 15 min at 25 °C. Upon centrifugation, the supernatant containing the purified recombinant SULT2B1a allozyme was collected and analyzed by 12% sodium dodecyl sulfate (SDS)–polyacrylamide gel electrophoresis (PAGE) in order to assess the purity of purified recombinant SULT2B1a allozyme. Protein concentrations of purified enzymes were determined by the Bradford protein assay method with bovine serum albumin as a standard^19^.

### Enzymatic assay for the sulfating activity of the purified recombinant SULT2B1a allozymes

The sulfating activity of the purified recombinant SULT2B1a allozymes towards pregnenolone was performed with [^35^S]-labeled PAPS. The standard assay mixture, with a final volume of 20 µL involving 50 mM HEPES buffer at pH 7.4, 1 mM DTT, the sulfonate donor (14 µM [^35^S]PAPS (15 Ci/mmol)), and the substrate (pregnenolone) or DMSO for the control. The reaction was initiated by adding 0.8 µg of the enzyme, allowed to proceed for 10 min at 37 °C, and terminated by heating at 98 °C for 3 min. Precipitates, formed in the heated reaction mixture, were cleared by centrifugation at 13,000 × g for 3 min. Then, 1 μl of the cleared reaction mixture was spotted on a thin-layer chromatography (TLC) plate, and the plate was subjected to TLC analysis using a solvent system containing *n*-butanol/isopropanol/formic acid/water in a ratio of 3:1:1:1 (by volume). Subsequently, the TLC plate was air-dried and autoradiographed using an X-ray film. The autoradiograph taken from the TLC plate was used to locate the radioactive spot corresponding to the sulfated product, and the located spot was cut from the TLC plate, eluted in 0.5 mL water, and mixed thoroughly with 2 ml of Ecolume scintillation liquid. The radioactivity was measured using a liquid scintillation counter. The results obtained were used to calculate the specific activity in the unit of sulfated product formed/min/mg of enzyme.

### Kinetic studies and statistical analysis

The SULT assay was performed using different concentrations of pregnenolone (0.05, 0.1, 0,15, 0.2, 0.3, 0.6, 1, 5, 10 µM) at pH 7.4 to determine the kinetic parameters for the wild-type SULT2B1a according to the procedure described above. Data obtained were analyzed based on Michaelis–Menten kinetics using GraphPad Prism^®^ (RRID: SCR_002798) v 6.0 software to calculate the *K*_m_ and *V*_max_ using non-linear regression curve.

## Results

### Identification and categorization of SNPs for the human SULT2B1 gene

**Figure 1:**
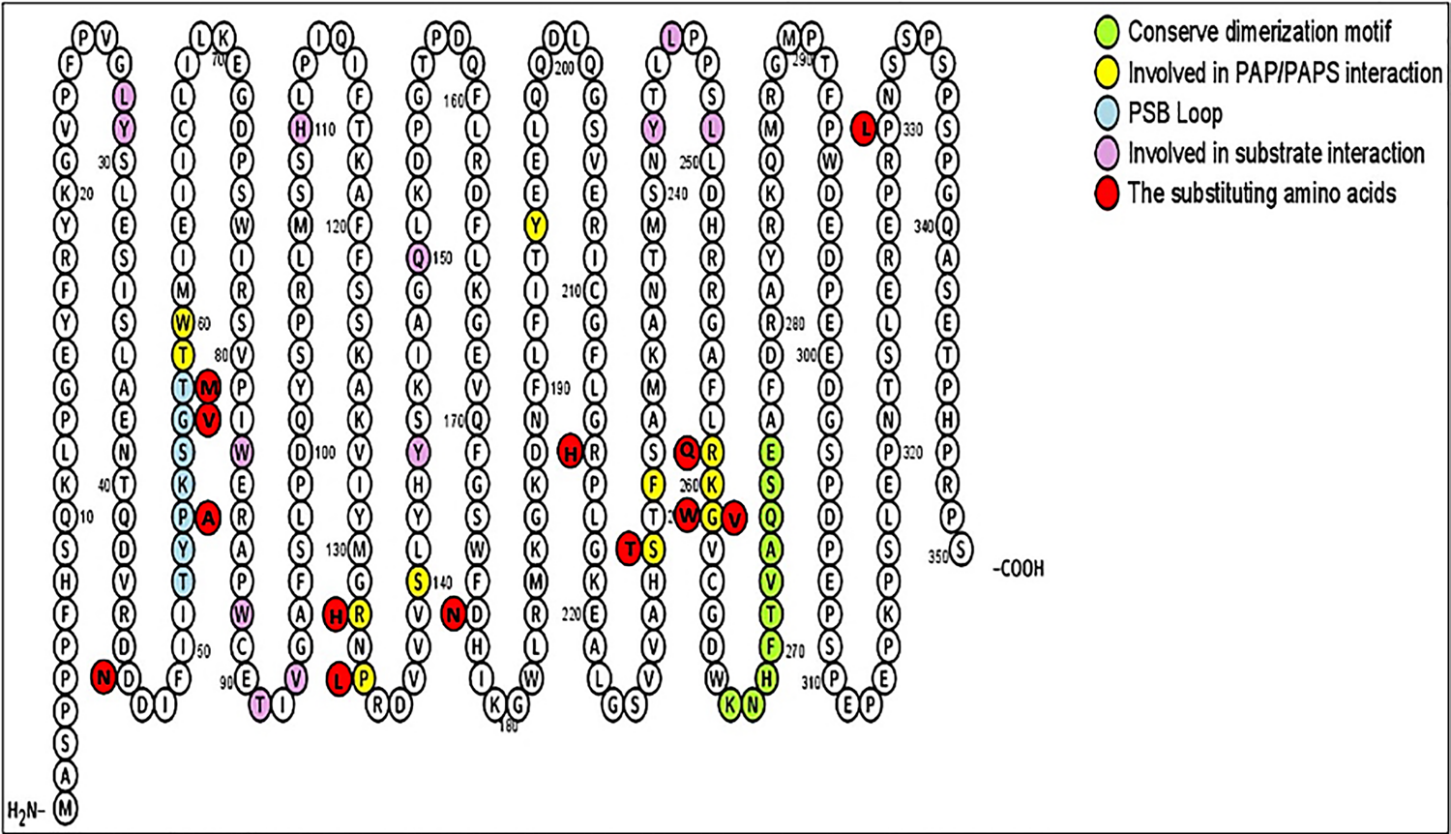
The amino acid sequence of the human SULT2B1a marked with residues/sequences reported to be involved in PAPS-binding, substrate-binding, dimerization-motif, and/or catalysis. The residues in yellow are those that are involved in PAPS-binding. Residues in green refer to those involved in the dimerization-motif. Residues in red are those involved in substrate-binding. Residues in purple refer to the substituting amino acids coded by the selected missense cSNPs. The figure was generated using protter tool^29^, a web tool for interactive protein feature visualization. *SULT2B1* Sulfotransferase family cytosolic 2B member 1, *PAPS* 3’-phosphoadenosine-5’-phosphosulfate, *cSNP* coding single nucleotide polymorphism.

Nucleotide sequence variations of human *SULT2B1* were analyzed by several online databases, including the NCBI, Ensembl Variation, and UniProt. Those sequence analyses detected a total of 20,459 *SULT2B1* SNPs, which were then categorized based on their location within the gene as being either coding (synonymous, nonsense, and non-synonymous [missense]) or non-coding (intronic or located in the 5’-UTR or 3’-UTR). In addition to the aforementioned database search, 16 *SULT2B1* SNPs were reported to be related to diseases by PubMed search (cf. Table 1). Of the identified SNPs, 326 were found to be missense cSNPs that result in amino acid changes in the protein product. These amino acid changes were further evaluated in terms of their locations relative to major enzyme features: the substrate binding site, the phosphosulfate binding (PSB) region, and PAPS-binding regions of SULT2B1, and the dimerization motif observed in other general SULT enzymes (Fig. 1)^14,20,21^. In this study, considering the chemical change of the mutated residues, 13 of the 326 missense SNPs were selected for further enzymatic investigation. Table 2 summarizes the selected amino acid changes, their locations, and the sense and antisense mutagenic primers designed for PCR amplification of corresponding *SULT2B1a* cDNAs.

**Table 1:** SULT2B1 SNPs that have been reported in scientific literature.

SNP ID	Location	Effect
rs4149455	Non coding region (intron variant)	Associated with reduced risks of esophageal squamous^22^
rs1052131	Coding region (synonymous codon)	
rs12460535	Non coding region (intron variant)	Associated with a reduced risk of prostate cancer progression^23^
rs2665582	Non coding region (intron variant)	
rs10426628	Non coding region (5’-UTR)	
rs3786749	Intron variant	Associated with colorectal cancer progression^24^
rs16982149	Coding region (missense mutation)	Cause changes in sulfating activity^25^
rs16982158		
rs16982169		
rs17842463		
rs3760806	Non coding region (5’-UTR)	Associated with an increased risk of colorectal cancer^26^
rs3760808	Non coding region (intron variant)	Associated with an increased risk of prostate cancer^27^
rs10424237		
rs762765702	Coding region (missense mutation)	Cause autosomal-recessive congenital ichthyosis in humans^28^
rs1114167424		
rs1114167426	Non coding region (splice donor)	

*SULT2B1* Sulfotransferase family cytosolic 2B member 1, *SNP* single nucleotide polymorphism, *UTR* untranslated region.

**Table 2:** List of the selected SULT2B1a cSNPs, sense and antisense mutagenic primers sets designed for the site-directed mutagenesis of the corresponding cDNAs encoding human SULT2B1a allozymes.

SNP code no	hSULT2B1a allozymes	Mutagenic primer nucleotides sequence
–	Asp46Asn	5’-ACCCAAGATGTGCGGGAC***A****AC*GACATCTTTATCATC-3’ 5’-GATGATAAAGATGTC*GT****T***GTCCCGCACATCTTGGGT-3’
rs777924668	Pro54Ala	5’-ATCTTTATCATCACCTAC***G****CC*AAGTCAGGCACGACC-3’ 5’-GGTCGTGCCTGACTT*GG****C***GTAGGTGATGATAAAGAT-3’
rs746398875	Gly57Val	5’-ATCACCTACCCCAAGTCA*G****T****C*ACGACCTGGATGATC-3’ 5’-GATCATCCAGGTCGT*G****A****C*TGACTTGGGGTAGGTGAT-3’
rs527454384	Thr58Met	5’-ACCTACCCCAAGTCAGGC*A****T****G*ACCTGGATGATCGAG-3’ 5’-CTCGATCATCCAGGT*C****A****T*GCCTGACTTGGGGTAGGT-3’
rs777140014	Arg132His	5’-AAGGTGATCTACATGGGC*C****A****C*AACCCCCGGGACGTT-3’ 5’-AACGTCCCGGGGGTT*G****T****G*GCCCATGTAGATCACCTT-3’
rs1114167424	Pro134Leu	5’-TCTACATGGGCCGCAAC*C****T****C*CGGGACGTTGTGGTCT-3’ 5’-AGACCACAACGTCCCG*G****A****G*GTTGCGGCCCATGTAGA-3’
rs16982158	Asp176Asn	5’-CAGTTTGGCTCCTGGTTC***A****AC*CACATTAAGGGCTGG-3’ 5’-CCAGCCCTTAATGTG*GT****T***GAACCAGGAGCCAAACTG-3’
rs16982169	Arg215His	5’-ATCTGTGGGTTCCTGGGC*C****A****T*CCGCTGGGCAAGGAG-3’ 5’-CTCCTTGCCCAGCGG*A****T****G*GCCCAGGAACCCACAGAT-3’
rs765224593	Ser229Thr	5’-GGCTCCGTCGTGGCACAC***A****CA*ACCTTCAGCGCCATG-3’ 5’-CATGGCGCTGAAGGT*TG****T***GTGTGCCACGACGGAGCC-3’
rs762765702	Arg259Gln	5’-CGTCGCGGGGCCTTCCTC*C****A****G*AAAGGGGTCTGCGGC-3’ 5’-GCCGCAGACCCCTTT*C****T****G*GAGGAAGGCCCCGCGACG-3’
rs774212320	Gly261Val	5’-GGGGCCTTCCTCCGGAAA*G****T****G*GTCTGCGGCGACTGG-3’ 5’-CCAGTCGCCGCAGAC*C****A****C*TTTCCGGAGGAAGGCCCC-3’
–	Gly261Trp	5’-GGGGCCTTCCTCCGGAAA***T****GG*GTCTGCGGCGACTGG-3’ 5’-CCAGTCGCCGCAGAC*CC****A***TTTCCGGAGGAAGGCCCC-3’
rs17842463	Pro330Leu	5’-CTGGAGCGTGAGCCCAGA*C****T****C*AACTCCAGCCCCAGC-3’ 5’-GCTGGGGCTGGAGTT*G****A****G*TCTGGGCTCACGCTCCAG-3’

*SULT2B1* Sulfotransferase family cytosolic 2B member 1, *cSNP* coding single nucleotide polymorphism.

*Italics show the codons to be mutated, and bold shows the substituted nucleotides.

### Expression and purification of recombinant human SULT2B1a allozymes

**Figure 2:**
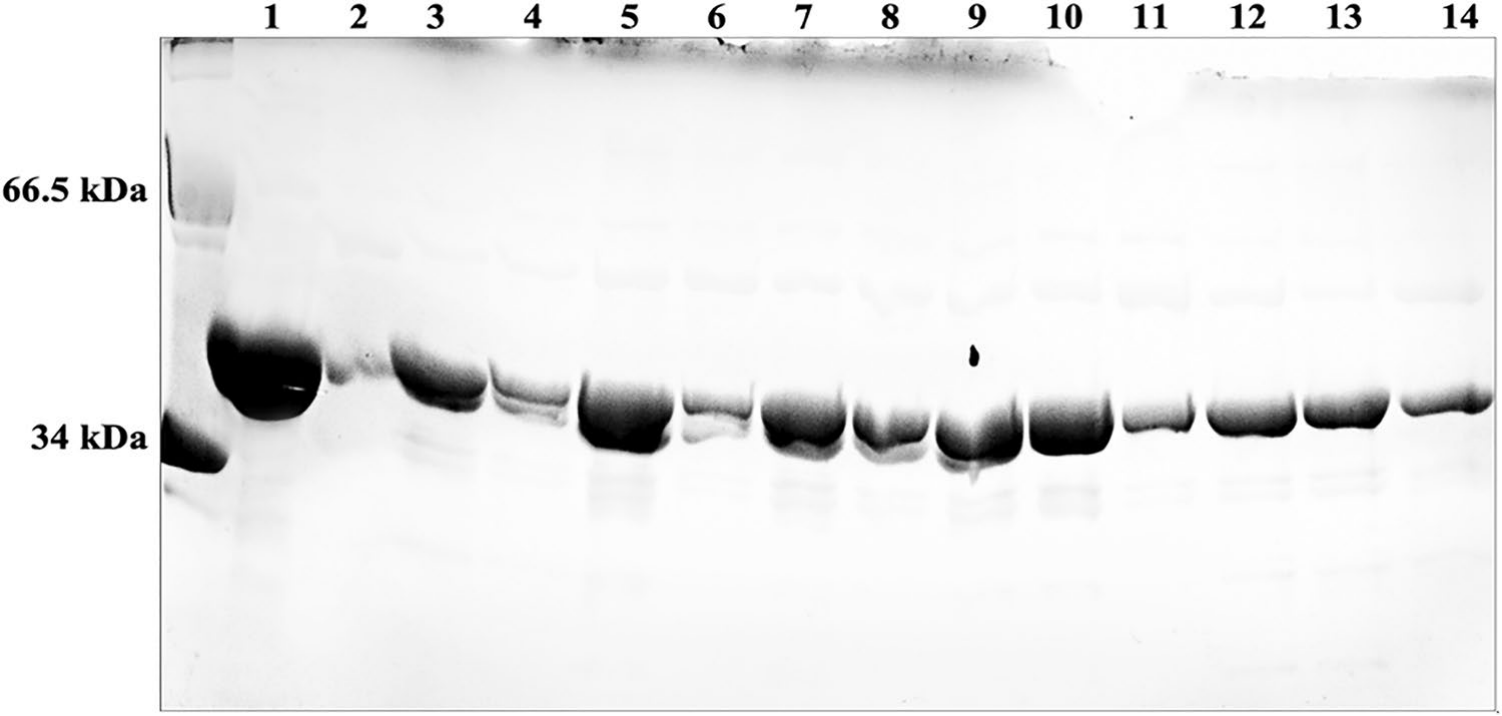
SDS gel electrophoretic pattern of purified wt-SULT2B1a and SULT2B1a allozymes. SDS-PAGE was performed on a 12% gel, followed by Coomassie blue staining. Lane * indicates the migrating positions of protein molecular weight markers co-electrophoresed. Samples analyzed in lanes 1 through 14 correspond to SULT2B1a-wt, P45A, T58M, P134L, R259Q, R132H, D176N, R215H, P330L, S229T, G57V, G261V, G261W, and D46N allozymes. *wt* wild-type, *SULT2B1* Sulfotransferase family cytosolic 2B member 1.

cDNAs encoding the 13 selected SULT2B1a allozymes were packaged in the pGEX-4 T-2 as described in the “Methods” section. The recombinant allozymes prepared were analyzed by SDS-PAGE and confirmed to be homogeneous and the high purity (cf. Fig. 2). For all 13 allozymes, the apparent molecular weights were comparable to the predicted molecular weight (~ 39.6 kD) of wild-type SULT2B1a (cf. lane 1 in Fig. 2).

### Kinetic study of pregnenolone sulfation by wild-type SULT2B1a

**Figure 3:**
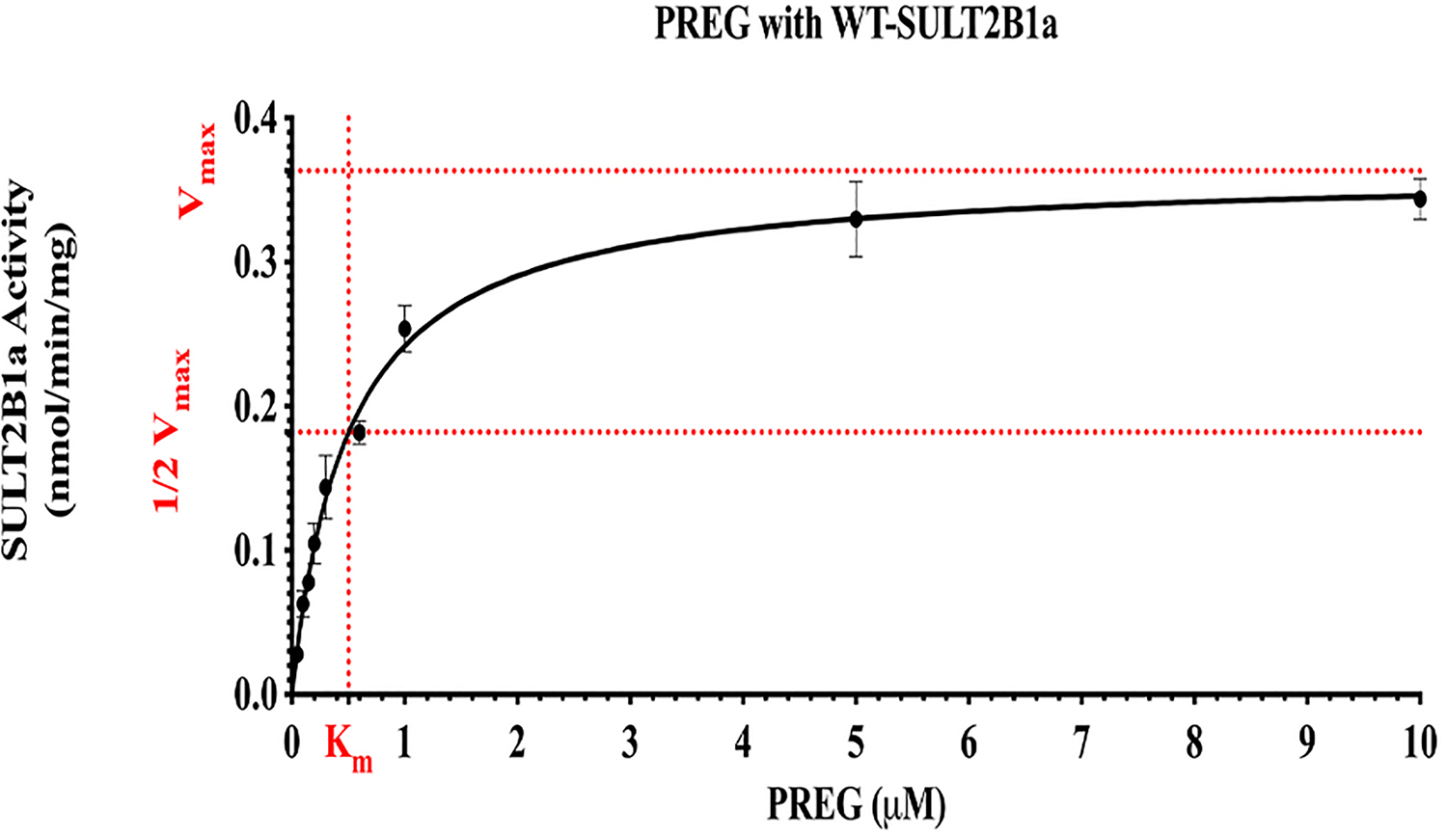
Concentration-dependent sulfation of pregnenolone by wild-type SULT2B1a. Data shown represent calculated mean ± standard deviation derived from three experiments. *wt* wild-type, *SULT2B1a* Sulfotransferase family cytosolic 2B member 1a, *PREG* pregnenolone.

The sulfating activity of wild-type SULT2B1a was evaluated using different concentrations of the substrate pregnenolone**,** as mentioned in the “Methods” section. Sulfation of pregnenolone by SULT2B1a appeared to follow the Michaelis–Menten kinetics up to a substrate concentration of 5 µM. *K*_m_ and *V*_max_ values determined were 0.50 ± 0.03 µM and 0.36 ± 0.01 nmol/min/mg, respectively (cf. Fig. 3).

### Sulfating activities of SULT2B1a allozymes with pregnenolone

**Figure 4:**
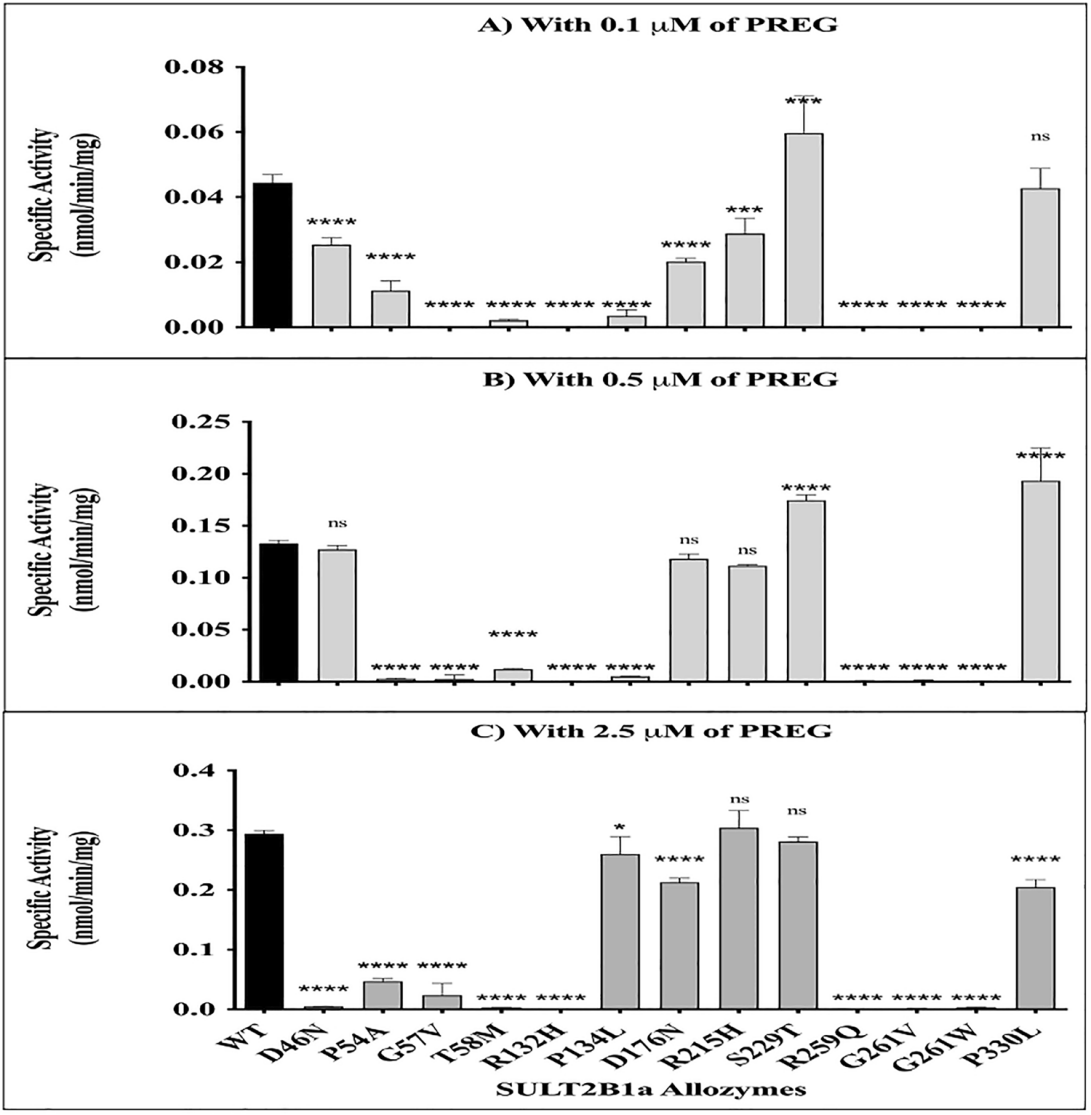
Specific activities of human SULT2B1a allozymes for the sulfation of pregnenolone at 0.1 µM (**A**), 0.5 µM (**B**), and 2.5 µM (**C**) concentrations. Data shown represent means ± standard deviations derived from three determinations. One-way ANOVA was performed in combination with Dunnett’s multiple comparison test. *SULT2B1* Sulfotransferase family cytosolic 2B member 1, *PREG* pregnenolone.

The sulfating activities of purified SULT2B1a allozymes toward pregnenolone were experimentally determined and compared to the wild-type enzyme. These assays utilized three different pregnenolone concentrations based on the results from the kinetic study of wild-type SULT2B1a: one concentration below the *K*_*m*_*,* one close to the *K*_*m*_, and one above the *K*_*m*_. Figure 4 depicts the results obtained from these comparative studies. Across all three substrate concentrations (0.1, 0.5, and 2.5 µM), the relative pregnenolone-sulfating activities of the 13 allozymes tested were generally consistent. Seven allozymes exhibited barely detectable sulfating activity at across all three concentrations: P54A, G57V, T58M, R132H, R259Q, G261V, and G261W. Of the remaining five allozymes, three (P134L, D176N, R215H) showed lower activity at 0.1 µM and similar activity at 2.5 µM, implicating that those three substitutions may weaken the affinity with pregnenolone. S229T and P330L showed slightly higher activity than the wild-type, implicating that two substitutions may increase the affinity with pregnenolone. The last allozyme, D46N, exhibited the sulfating activity comparable to the wild-type at 0.5 µM, but the activity at 2.5 µM was reduced to one-tenth of its activity at 0.5 µM. D46N substitution may alter the Michaelis–Menten kinetics of wild-type to the substrate inhibition kinetics.

## Discussion

The endogenous steroid pregnenolone is the precursor intermediate from which most other steroid hormones are derived. Sulfation of hydroxysteroids is predominantly carried out by SULT2 members, and SULT2B1a has been shown to favor pregnenolone as a substrate compared to other members of the *SULT2* family^30–32^. The current study represents the first systematic investigation of the pregnenolone sulfating activity of SULT2B1a allozymes, including their substrate affinity compared to the wild-type enzyme. A systematic database search identified missense cSNPs that occur in the human *SULT2B1a* gene. A total of 13 missense cSNPs were selected to evaluate their sulfating activities toward pregnenolone. The assays with three concentrations of pregnenolone, selected with reference to the *K*_m_ of the wild-type enzyme, clearly indicated the differential sulfating activities and affinities of allozymes with pregnenolone.

Several studies in the literature provide information regarding what parts of SULT2B1a are essential for its sulfating activity^32,33^. Mutational analysis of human SULT2B1a has revealed that removing either 53 amino acids from the long carboxyl-terminal end or eight from the amino-terminal end does not affect its pregnenolone sulfotransferase activity^32^. While no crystal structure is available for SULT2B1a specifically, one has been reported for the alternative splice variant, SULT2B1b^20^. Regarding the enzyme’s internal structure, the PSB loop is composed of the conserved amino acid residues ^52^TYPKSGT^58^, of which the residues Lys^55^, Ser^56^, Gly^57^, and Thr^58^ are involved in binding the 5’-phosphate of PAP/PAPS. Meanwhile, the residues Arg^132^, Ser^140^, Arg^259^, Lys^260^, and Gly^261^ are involved in binding its 3’-phosphate. The adenine group of PAP also interacts with the amino acid residues Trp^75^, Tyr^195^, Ser^229^, and Phe^246^, while the catalytic activity of the enzyme is due to His^110^^20^. Notably, consistent with expectations regarding impacts on enzyme activity, the majority of the SULT2B1a allozymes examined in this study involve amino acid changes either within or close to these important structural elements.

In the cases of allozymes with little or no detectable sulfating activity, six directly involve the abovementioned structural residues (G57V, T58M, R132H, R259Q, G261V, and G261W), while the other two involve nearby residues (D46N and P54A). Substitution of these critical amino acids may have weakened the interaction between SULT2B1a and its essential cofactor PAPS, and consequently abolished or dramatically decreased allozyme activity toward pregnenolone. Meanwhile, the other five tested allozymes (P134L, D176N, R215H, S229T, and P330L) exhibited sulfating activities comparable to the wild-type at 2.5 µM. These included two substitutions impacting key structural residues or nearby residues (P134L and S229T), and three involving residues relatively distant from known essential elements (D176N, R215H, and P330L). For P134L, it is probable that the substitution of this proline (a turn-inducing residue) with leucine (a non-turn-inducing residue) affected the conformation of the protein backbone, thus impairing (but not preventing) hydrogen bonding between the NH_2_ of nearby Arg^132^ and the oxygen atom of the 3’-phosphate in PAPS^20,28^. By contrast, despite directly impacting an essential residue, the substitution S229T resulted in an allozyme with 96% of the activity of wild-type. This outcome is consistent with the chemical similarity of serine and the substituted threonine (both being polar and hydrophilic amino acids).

Meanwhile, the allozyme with substitution D176N did not replace a known key residue, yet exhibited reduced sulfating activity. The original aspartic acid (D) and the substituted asparagine (N) have different chemical properties, which likely relates to the reduced activity and affinity of this allozyme. Distinct from any other tested allozyme, the R215H substitution yielded sulfating activity slightly lower than the wild-type at 0.1 µM and no effect at higher two concentrations. In this case, as the original and substituted amino acids featured similar chemical properties (both being positively charged), the substitution may weakly affect the affinity but not the maximum velocity. Finally, the P330L substitution resulted in an allozyme with activity 30% lower than that of the wild-type at 2.5 µM. This allozyme replaced a turn-inducing proline with non-turn-inducing leucine; as proline residues are important for the conformation of the protein backbone, it would be reasonable to expect this polymorphism to greatly impact activity. However, previous mutational analysis of SULT2B1a showed the removal of 53 amino acids from the long carboxyl-terminal end, including P330, to have no effect on its pregnenolone sulfotransferase activity^32^, implying relatively less structural import for this residue. These observations are consistent with the activity data of SULT2B1b allozymes toward pregnenolone previously reported^16^, suggesting that single nucleotide polymorphisms on the common region of the *SULT2B1* gene may have a similar effect on the sulfating activity of the two splice variants of SULT2B1 toward pregnenolone.

It should be noted that two non-synonymous coding cSNPs of the SULT2B1b variant in the *SULT2B1* gene, c.446C > T (P149L) and c.821G > A (R274Q), have been previously reported to be involved in autosomal recessive ichthyosis (ARCI)^28^. Impairment of cholesterol metabolism, including accumulation of cholesterol or cholesterol sulfate, has been observed in patients with ARCI^28,34^. The SULT2B1b variant, responsible for cholesterol sulfation, has been recognized to be involved in ARCI. The two cSNPs mentioned above may also generate two SULT2B1a allozymes, P134L and R259Q. In our study, SULT2B1a wild-type and all selected allozymes have been analyzed for sulfating activity toward cholesterol. As expected, all have exhibited no detectable sulfating activity toward cholesterol while both P134L and R259Q allozyme showed reduced pregnenolone-sulfating activity. Within the differentiating sequence, residues I20 and I23 are primarily responsible for SULT2B1b’s high cholesterol sulfating activity^32^. In particular, residue I20 forms positive Van der Waal’s interactions with the long chain C17 cholesterol atom; such interactions are negligible in the binding of pregnenolone^20^. Therefore, secondary structure in the amino terminus is responsible for determining the substrate selectivity of SULT2B1 isoforms (*a* and *b*). Therefore, ARCI patients with the aforementioned two cSNPs may result in the loss of SULT2B1a function, as well as SULT2B1b, implying that less sulfation of pregnenolone and DHEA possibly impair the neurosteroid physiology.

## Conclusions

This study is the first to provide information concerning the effects of *SULT2B1* cSNPs on the pregnenolone-sulfating activity of corresponding SULT2B1a allozymes. Of the 13 tested allozymes, eight exhibited minimal to no detectable sulfating activity (D46N, P54A, G57V, T58M, R132H, R259Q, G261V, and G261W), while the other five exhibited differential sulfating activity in comparison with the wild-type (P134L, D176N, R215H, S229T, and P330L). Thus, this research clearly demonstrates a significant effect of some *SULT2B1* genetic polymorphisms on corresponding allozyme activity, and supports the possibility that individuals harboring different *SULT2B1a* genotypes may have differential capacity in sulfating pregnenolone. Additional research may help in predicting the risk of some *SULT2B1a*-associated diseases, as well as assist the design of personalized regimens of relevant drugs for individuals with different *SULT2B1a* genotypes.

## Data Availability

The datasets generated during and/or analyzed during the current study are available in the Figshare repository, DOI: https://figshare.com/s/44e0183a2db3dcff627f. Data are available under the terms of the Creative Commons Zero “No rights reserved” data waiver (CC0 1.0 Public domain dedication).
