# Author Correction: The role of genetic polymorphisms in the sulfation of pregnenolone by human cytosolic sulfotransferase SULT2B1a

**DOI:** 10.1038/s41598-025-93641-x

**Published:** 2025-03-27

**Authors:** Eid Alatwi, Ahsan F. Bairam

**Affiliations:** 1 Department of Pharmacology, College of Pharmacy, Jouf University, Sakaka, Al-Jouf Region Saudi Arabia; 2 Department of Pharmacology and Experimental Therapeutics, College of Pharmacy and Pharmaceutical Sciences, University of Toledo Health Science Campus, Toledo, OH 43614 USA; 3 Department of Clinical Pharmacy, College of Pharmacy, University of Kufa, Kufa Street, Najaf, 540011 Iraq

Correction to: *Scientific Reports* 10.1038/s41598-024-56303-y, published online 05 April 2024

The original version of this Article contained an error in Affiliation 1, which was incorrectly given as ‘Department of Pharmacology, College of Pharmacy, Aljouf University, Aljouf, Saudi Arabia’. The correct affiliation is listed below:

Department of Pharmacology, College of Pharmacy, Jouf University, Sakaka, Al-Jouf Region, Saudi Arabia.

The original Article has been corrected.

